# The relationship between cholinergic system brain structure and function in healthy adults and patients with mild cognitive impairment

**DOI:** 10.1038/s41598-021-95573-8

**Published:** 2021-08-09

**Authors:** Jessica Peter, Isabella Mayer, Thomas Kammer, Lora Minkova, Jacob Lahr, Stefan Klöppel, Michel J. Grothe, Michael Orth

**Affiliations:** 1 University Hospital of Old Age Psychiatry and Psychotherapy, Bern University, Bolligenstraße 111, 3000 Bern 60, Switzerland; 2 Department of Neurology, Ulm University, Ulm, Germany; 3 Section for Neurostimulation, Department of Psychiatry, Ulm University, Ulm, Germany; 4 Department of Psychiatry and Psychotherapy, Faculty of Medicine, Freiburg University, Freiburg im Breisgau, Germany; 5 Unidad de Trastornos del Movimiento, Servicio de Neurología Y Neurofisiología Clínica, Instituto de Biomedicina de Sevilla (IBiS), Hospital Universitario Virgen del Rocío/CSIC/Universidad de Sevilla, Seville, Spain; 6 Neurozentrum Siloah, Worbstr. 316, Gümligen, 3073 Bern, Switzerland

**Keywords:** Ageing, Neural circuits, Sensory processing, Thalamus

## Abstract

We assessed the structure–function relationship of the human cholinergic system and hypothesized that structural measures are associated with short-latency sensory afferent inhibition (SAI), an electrophysiological measure of central cholinergic signal transmission. Healthy volunteers (n = 36) and patients with mild cognitive impairment (MCI, n = 20) underwent median nerve SAI and 3T structural MRI to determine the volume of the basal forebrain and the thalamus. Patients with MCI had smaller basal forebrain (*p* < 0.001) or thalamus volumes (*p* < 0.001) than healthy volunteers. Healthy SAI responders (> 10% SAI) had more basal forebrain volume than non-responders (*p* = 0.004) or patients with MCI (*p* < 0.001). More basal forebrain volume was associated with stronger SAI in healthy volunteers (*r* = 0.33, *p* < 0.05) but not patients with MCI. There was no significant relationship between thalamus volumes and SAI. Basal forebrain volume is associated with cholinergic function (SAI) in healthy volunteers but not in MCI patients. The in-vivo investigation of the structure–function relationship could further our understanding of the human cholinergic system in patients with suspected or known cholinergic system degeneration.

## Introduction

The cholinergic brain in mammals anatomically consists of the four main regions basal forebrain, the brainstem pedunculo-pontine and lateral dorsal tegmental nuclei, a subset of thalamic nuclei as well as the striatum and cortex, that contain cholinergic interneurons^1^. The cholinergic neurons projecting to the cortex cluster in the basal forebrain (Ch1 to Ch4), and their projections modulate functionally and spatially distinct regions of the neocortex as well as the thalamic reticular nucleus, the hippocampus and the amygdala^1–3^. Basal forebrain volumes decline in ageing humans and are severely reduced in patients with dementia due to Alzheimer’s disease (AD), a neurodegenerative disease associated with a loss of cholinergic brain structure and their cholinergic projection neurons^4–6^.

An important question is how cholinergic brain structure relates to brain function that is influenced by cholinergic projection neurons. Cholinergic pathways can be examined in-vivo using short-latency sensory afferent inhibition (SAI), a transcranial magnetic stimulation (TMS) paradigm that non-invasively measures the effect on motor cortex (M1) excitability of a preceding, conditioning sensory afferent electrical stimulus given to a peripheral mixed nerve above its motor threshold^7^. If the interval between the peripheral nerve conditioning stimulus and the motor cortex TMS pulse is slightly longer than the N20 latency of somatosensory evoked potentials, the motor cortex becomes less excitable. The amount of SAI is reduced by the administration of scopolamine, a muscarinic acetylcholine receptor antagonist^8^, indicating a contribution of cholinergic signal transmission to SAI. Basal forebrain and/or thalamus grey matter volumes can be extracted from structural MRI^9^. Basal forebrain and/or thalamus volumes could relate to the cholinergic influence on functional connections to cortical and subcortical structures including those serving sensory-motor integration^10^.

We hypothesized that the structural measures of the cholinergic brain regions are associated with the response to SAI. We expected to find less SAI in healthy people with lower volumes of either basal forebrain or thalamus. In addition, we expected less SAI in patients in the prodromal stage of AD (i.e., mild cognitive impairment; MCI) since reduced SAI has been reported in AD^11^. We also expected to find less basal forebrain volume in patients with MCI since atrophy in this population is well recognized^9^.

## Material and methods

### Participants

We included 36 healthy volunteers (n = 12 Ulm and n = 24 Freiburg) and 20 patients with mild cognitive impairment (MCI) from the Centre for Geriatric Medicine and Gerontology at the University Medical Centre Freiburg. All patients with MCI needed to fulfil criteria for a diagnosis of ‘MCI due to AD’ according to established criteria^12^. This required at least one cognitive function to be below 1.5 SD on the CERAD neuropsychological battery^13^. In addition, they needed to (a) report a cognitive complaint, (b) show no impairment in activities of daily living, (c) no dementia but (d) signs of neuronal injury (i.e., hippocampal or medial temporal atrophy by volumetric measures of visual rating).

Healthy volunteers were included in the study if they were 18 years or older and had no major psychiatric, neurological or medical disorder or a history of epilepsy or severe head injury, drug or alcohol abuse, contra-indications to TMS, or medication. In addition, they were included when no signs of cognitive impairment were found (i.e., the Montreal Cognitive Assessment score was ≥ 23 as recommended by Carson and colleagues^14^). No participant experienced any unwanted effects during or after the experiments. The Ethics Committees of Freiburg University and Ulm University approved the study, which we conducted according to the Declaration of Helsinki. All participants gave written informed consent before the study.

### Magnetic resonance imaging

High-resolution structural MRI data were acquired using 3T scanners (Magnetom ALLEGRA in Ulm; Tim Trio in Freiburg, Siemens, Erlangen, Germany). The MRI parameters of the three-dimensional magnetization-prepared rapid gradient-echo (3D-MPRAGE) sequences in Ulm were as follows: flip angle 9°, TE = 3.67 ms; TR = 2200 ms; slice thickness = 1 mm; resolution = 1.0 × 1.0 × 1.0 mm^3^. The parameters applied in Freiburg were as follows: flip angle of 12°, TE = 2.15 ms, TR = 2200 ms, slice thickness of 1 mm, resolution = 1.0 × 1.0 × 1.0 mm^3^.

MRI data were pre-processed using the Computational Anatomy Toolbox (CAT12; http://www.neuro.uni-jena.de/cat) and Statistical Parametric Mapping (SPM12, Welcome Trust Centre for Neuroimaging) implemented in Matlab R2016b (MathWorks, Natick, MA, USA) following the standard default procedure suggested in the manual. Briefly, pre-processing consisted of tissue class segmentation, registration to a standard template, spatial normalization of the grey matter maps, modulation of grey matter voxel values to preserve the amount of volume present before normalization, and final data quality check. The standard pre-processing pipeline does not include any correction for head size or volume, which is why we estimated the total intracranial volume (TIV) of each participant as the total sum of all segmented tissue classes and used it as a covariate in all statistical analyses including MRI data.

### Volumes of the basal forebrain cholinergic system and the thalamus

Volumes of the thalamus were determined using the ‘Estimate mean values inside ROI’ function in the CAT12 toolbox. For each participant CAT12 automatically performed brain structure parcellations and estimated tissue volumes of regions of interest (ROI) using the Neuromorphometrics brain atlas (Neuromorphometrics, Inc.) during the pre-processing step. Volumes of the basal forebrain were determined using a mask obtained from a recent functional parcellation study of a cytoarchitectonically-defined cholinergic basal forebrain ROI based on combined histology and post-mortem MRI^15^. We combined volumes of the left and the right hemisphere for statistical analyses.

### Electromyography recordings

We recorded surface electromyograms (EMG) from the abductor pollicis brevis (APB) muscle of the dominant hand using silver/silver-chloride disc surface electrodes (1 cm diameter) in a belly tendon montage. The EMG signal was amplified and filtered (20 Hz-2 kHz). Data (sampling rate 5 kHz) were digitized for off-line analysis.

### Transcranial magnetic stimulation

Participants were seated in a comfortable chair. They were asked to relax as much as possible. Magnetic stimuli were given with a hand-held figure-of-eight coil (outer winding diameter 9 cm) connected to a High Power Magstim 200 stimulator (Magstim Co., Whitland, Dyfed, UK). This stimulator generates a magnetic pulse with monophasic waveform and induces a current in the brain with posterior-anterior flow when the coil handle is positioned at an angle of 45° pointing backwards. The optimal spot for APB stimulation was marked with a felt pen.

### Resting motor threshold

Resting motor threshold (RMT) was defined as the minimum intensity (in % stimulator output) needed to evoke a motor-evoked potential (MEP) of > 50 µV in 5 out of 10 consecutive trials in the relaxed APB. The threshold was approached from above threshold in steps of 1% stimulator output. Once no MEP could be elicited the intensity was increased in steps of 1% stimulator output until a minimal MEP was observed. This intensity was taken as motor threshold.

### Short-latency sensory afferent inhibition (SAI)

SAI of the motor cortex was examined as previously described^7^. In brief, an MEP of ~ 1 mV peak-to-peak amplitude was elicited in the APB by TMS. This was preceded by a supra-threshold electrical stimulus given to the median nerve through bipolar surface electrodes with the cathode proximal to the anode. Median nerve stimulation was set at just above motor threshold to elicit an M-wave with about 500 µV peak-to-peak amplitude. The inter-stimulus intervals were related to the individual N20 latency in somatosensory evoked potentials^16^ in Ulm (N20 + 2 and N20 + 4) while in Freiburg fixed inter-stimulus interval of 20 and 21 ms were used^7^.

With an inter-trial interval of 5 s 20 unconditioned stimuli (TMS stimulation alone) as well as 10 conditioned stimuli (TMS preceded by peripheral nerve stimulation) at each inter-stimulus interval were given in randomized order. Using visual feedback, trials recorded while the participants contracted the hand muscles were excluded online. No trials were excluded in the offline analysis. The peak-to-peak amplitude was measured, and the average amplitude of the conditioned MEP was expressed in percent of the average amplitude of the unconditioned MEP. The minimum response mean MEP amplitude at either of the inter-stimulus intervals was taken as the maximum inhibition of corticospinal excitability. We defined a response as an amount of inhibition of at least 10%^16^.

### Data analysis

We used univariate or multivariate ANOVA to examine whether there were differences in demographic, electrophysiology, or structural imaging data (corrected for age and TIV) between the combined healthy volunteers from the Ulm and Freiburg sites, and MCI participants. For the analysis of frequencies (e.g., sex or SAI response) we applied either *Χ*^2^ or Fisher`s exact test. For associations between structural imaging data and electrophysiology data we used partial correlations while controlling for age and TIV. We used SPSS (version 26.0; IBM Inc.; USA) for statistical analyses with *p* < 0.05 considered statistically significant.

### Ethics approval

The Ethics Committees of Freiburg University and Ulm University approved the study.

### Consent to participate

All participants gave written informed consent before the study.

## Results

The healthy volunteers from the Ulm site were younger than the healthy volunteers from the Freiburg site or the patients with MCI (*F*_(2,53)_ = 82.34, *p* < 0.001; Table 1); the healthy Freiburg volunteers and the MCI patients were of similar ages. Thus, we included age as a covariate in all statistical analyses. Patients with MCI scored lower in the MoCA than healthy volunteers (*F*_(2,53)_ = 39.04, *p* < 0.001; Table 1). Post-hoc tests revealed that patients with MCI achieved lower scores than both healthy controls from Ulm (*p* < 0.001) and Freiburg (*p* < 0.001). Healthy volunteers from Ulm and Freiburg were not statistically different in their MoCA scores.

**Table 1:** Socio-demographic data of the sample and group comparison for cholinergic structure and function. We used multivariate ANOVA for group comparison if not stated otherwise.

	Ulm (HC)		Freiburg (HC)		Freiburg (MCI)		*p*-value
	Mean	SD	Mean	SD	Mean	SD	
n (f/m)	12 (7/5)		24 (17/7)		20 (11/9)		0.55 (Fisher)
Age (years)	50.3	4.8	70.4	5.6	73.5	4.9	< 0.001
MoCA (0–30)	28.4	1.1	27.4	1.7	22.8	2.6	< 0.001
Median nerve motor threshold (mA)	8.2	1.9	9.4	2.0	8.5	1.9	0.12
Resting motor threshold (% stimulator output)	46.7	10.4	54.2	10.7	52.2	11.2	0.63
SAI max inhibition	0.58	0.2	0.58	0.3	0.60	0.3	0.99
SAI responder (y/n)	10/2		20/4		15/5		0.83 (Fisher)
Basal forebrain volume (ml)	3.70	0.5	3.54	0.3	3.31	0.5	< 0.001
Thalamus volume (ml)	8.97	0.9	8.77	1.0	7.51	1.0	< 0.001

*SD* Standard deviation, *HC* Healthy controls, *MCI* Mild Cognitive Impairment, *f/m* female/male, *MoCA* Montreal Cognitive Assessment, *SAI* Short-latency sensory afferent inhibition, *y/n* number yes/number no.

### Structural data

**Figure 1:**
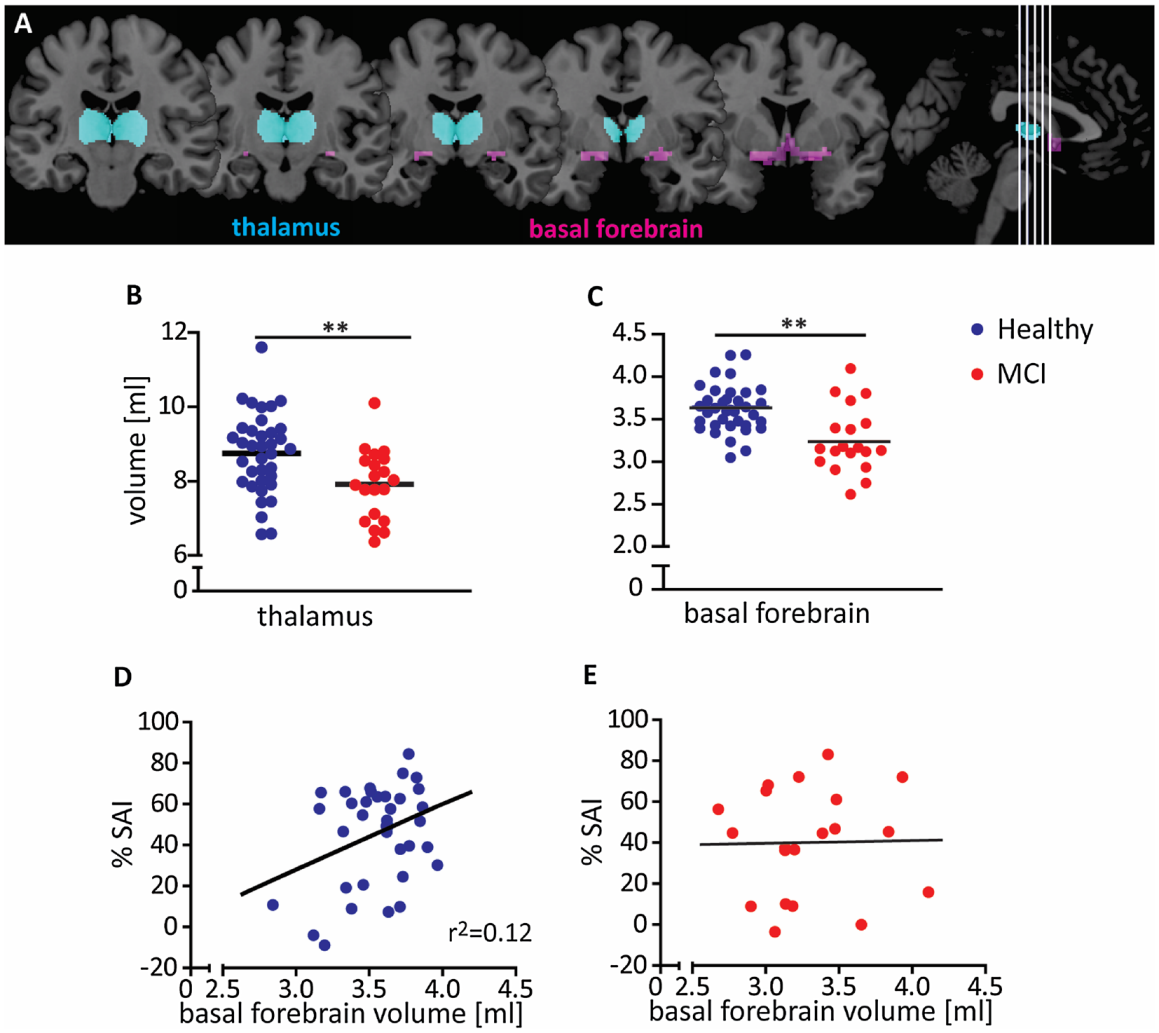
(**A**) Anatomic representation of the basal forebrain cholinergic system (pink) and the thalamus (turquoise) regions of interest. Thalamus (**B**) and basal forebrain volumes (**C**) were smaller in patients with mild cognitive impairment (n = 20; red) than in healthy volunteers (n = 36; blue). In healthy volunteers, a larger basal forebrain was associated with stronger short-latency sensory afferent inhibition (SAI) (**D**) while there was no association in MCI patients (**E**). All volume data were corrected for total intracranial volume. **p < 0.01.

We first examined basal forebrain and thalamus volumes as important regions of the cholinergic brain (Fig. 1A). Patients with MCI had significantly smaller basal forebrain (*F*_(1, 52)_ = 10.01, *p* = 0.003, Fig. 1B) or thalamus volumes (*F*_(1, 52)_ = 11.18, *p* = 0.002; Table 1, Fig. 1C) than the combined healthy volunteers. Post-hoc tests revealed that those differences existed not only in the combined healthy volunteer group but also separately between patients with MCI and healthy volunteers from Ulm (*p* < 0.05) or healthy volunteers from Freiburg (*p* < 0.01). There were no significant volume differences between Ulm and Freiburg for healthy volunteers. A larger volume of the basal forebrain was associated with a larger volume of the thalamus, when controlling for TIV (*r*_(52)_ = 0.41, *p* = 0.002).

### Electrophysiology

We next examined SAI as a functional measure of the cholinergic system^11^. The SAI maximum inhibition was similar in healthy volunteers and patients with MCI (Ulm, 43.68% ± 20.13; Freiburg, 42.49% ± 27.10; MCI, 38.17% ± 26.57) and so was the number of responders to SAI (Responder/Non-responder: Ulm, 10/2; Freiburg 20/4; MCI 15/5). Age was not significantly associated with SAI, and neither was there a statistical difference between men and women.

### Relationship between structure and function

We went on to ask whether there was a relationship between the structural and the functional data of the cholinergic brain. For healthy volunteers, we found that responders to SAI had significantly more basal forebrain volume than SAI non-responders (*F*_(1, 32)_ = 7.61, *p* = 0.01). In addition, more volume of the basal forebrain was associated with stronger SAI (*r*_(36)_ = 0.34, *p* = 0.04, Fig. 1D). In contrast, thalamus volumes were similar in SAI responders and non-responders and did not correlate with SAI as a continuous measure. Basal forebrain volumes in healthy SAI responders were significantly higher than in the MCI group (*F*_(1,41)_ = 9.89, *p* = 0.003), but basal forebrain volumes of the healthy SAI non-responders were similar to those of the MCI group.

In patients with MCI, we found no significant relationship between SAI response and basal forebrain volume or thalamus volume (Fig. 1E). Their basal forebrain, or thalamus, volumes were similar to those of the healthy SAI non-responders.

## Discussion

In the current study we examined the relationship between volume of the basal forebrain, the pacemaker of the cholinergic system, and short-latency sensory afferent inhibition (SAI), an electrophysiological paradigm used to measure cholinergic brain function. In healthy volunteers SAI non-responders had significantly less basal forebrain volume than those that responded well, and at an individual participant level more basal forebrain volume was associated with better cholinergic function. Basal forebrain volumes may become smaller with advancing age^17^. However, age did not explain the difference in SAI response associated with basal forebrain volumes. Lower basal forebrain volume in SAI non-responders may therefore, independent of age, reflect a lower density of cholinergic projections to structures thought to be involved in the regulation of motor cortex excitability. In the SAI paradigm, the influence on M1 excitability of the sensory-afferent conditioning volley could be mediated by direct thalamo-cortical projections to M1 or via connections from the somatosensory cortex (S1) to M1. Basal forebrain projections reach cortical areas including M1 and S1, at least in mice^18^ in which they coordinate the activity of these areas with associated regions. This contributes to the regulation of cognition and motor command as basal forebrain projections influence functionally and spatially selective cellular signaling and synaptic network function^1^ and were shown to have functional connections to cortical and subcortical structures in humans using resting state fMRI^10^. An increase of acetylcholine in the basal forebrain increases the cortical coding capacity as was shown for the visual cortex in mice^19^. Important for sensory-motor integration that is being probed with SAI, cholinergic modulation of neuronal function differs between the different cortical layers of the somatosensory cortex^20^. This includes the influence of acetylcholine on the responsiveness of cells in layer 4 of S1 that receives many inputs including from the thalamus. While it remains unclear which neuronal pathways SAI requires it is conceivable that, physiologically, there is variability in how different people process sensory afferent inputs to S1. If this were influenced by acetylcholine then it would be tempting to speculate that such differences depend, at least in part, on the density of cholinergic projections to S1 from the basal forebrain.

The thalamus is a key relay station in sensory-afferent pathways to the somatosensory cortex. The contribution to shaping cortical motor command extends beyond cholinergic basal forebrain projections to the cortex and includes the cholinergic projections to e.g., the thalamic reticular nucleus, an important regulator of thalamic function rich in receiving cholinergic projections from the basal forebrain^21^. We therefore examined the relationship of cholinergic function (i.e., SAI) to thalamus volumes. There was no association of SAI with thalamus volumes. Our analyses indicate, therefore, that the basal forebrain volume plays a greater part in predicting a response to SAI than the thalamus volume.

We then extended our analyses to include patients with MCI given that the cholinergic brain plays an important role in the pathogenesis of MCI and AD. In our MCI patients, as expected, basal forebrain volumes were smaller than in the healthy volunteers^17^. This agrees with the notion that the loss of basal forebrain volume precedes the cortical expression of AD degeneration^22,23^. However, unlike in healthy volunteers, there was no relationship between the structural measure of the cholinergic brain and SAI. In the MCI group, the amount of SAI, and the number of responders and non-responders were similar to healthy volunteers even though the basal forebrain, or thalamus, volumes in all MCI participants were smaller to a degree seen in healthy SAI non-responders. Given the data in our healthy volunteers one would have expected a greater number of SAI non-responders in the MCI group, in particular in light of the reports on reduced SAI in patients with AD^8^. This was not the case suggesting that the structure–function relationship in healthy individuals differs from that in MCI patients. It is possible that other factors that influence SAI become more important in order to maintain function as long as possible as cholinergic input to thalamus and cortex changes in the wake of basal forebrain degeneration. However, even in patients with AD a reduction of SAI is not a consistent finding^24^ suggesting that SAI is highly variable. Although at a population level there may be a reduction of SAI given the number of studies that have reported it in AD, the relationship is not clear and acetylcholine modulation may be more relevant in some individuals than others independent of whether they are healthy or affected by a neurodegenerative process involving the basal forebrain.

Our study has several limitations. Even though the number of participants was higher than in many other studies, it is possible that in light of the substantial within-participant variability of SAI the power to detect subtle effects at a participant level is not high enough with the number of participants we included^25^. We already aimed to increase the number of participants by combining two cohorts from two different study sites. While the difference in age between the two cohorts is a limitation, their basal forebrain, or thalamus, volumes were similar as was the SAI response suggesting that age, MRI protocols or using either inter-stimulus intervals according to the individuals’ SEP N20 latency or fixed inter-stimulus intervals did not substantially influence the results in accord with current methodological considerations^26^. A further limitation could be that with current MRI methodology we could not obtain structural information from individual thalamic nuclei so that we could not differentiate the thalamic reticular nucleus that receives cholinergic input or the ventral posterior nucleus as an important relay nucleus for somatosensory afferents. If, for instance, either of these nuclei played a prominent role in sensory afferent signal transmission involved in SAI while the remaining thalamus did not that specific contribution may have been diluted by using a volume measure of the whole thalamus. The same goes for midbrain cholinergic regions such as the pedunculopontine and laterodorsal tegmental nuclei that influence striatal function^27^.

## Conclusion

Taken together we show that structural properties of the basal forebrain, the pacemaker of the cholinergic brain, are associated with the response to SAI, an electrophysiological measure of cholinergic brain function. The in-vivo investigation of the structure–function relationship could further our understanding of the human cholinergic brain. A better understanding in healthy individuals may eventually have potential in the assessment of patients with suspected or known degeneration of the cholinergic brain, in which SAI alone may not suffice as biomarker^24^.

## Data Availability

Data will be made available upon request.
